# Insulin Resistance and Chronic Hepatitis C: Relationship With Serum Iron and Hepcidin

**DOI:** 10.7759/cureus.12349

**Published:** 2020-12-28

**Authors:** Maria Gill, Misbah Ul Qamar, Faiza Ikram, Shaheena Naz, Hina Sadaf, Zobiah Hafeez

**Affiliations:** 1 Physiology, CMH Multan Institute of Medical Sciences, Multan, PAK; 2 Physiology, Akhtar Saeed Medical and Dental College, Lahore, PAK; 3 Physiology, Avicenna Medical College, Lahore, PAK; 4 Physiology, Azra Naheed Medical College, Lahore, PAK; 5 Physiology, University of Lahore, University College of Medicine and Dentistry, Lahore, PAK

**Keywords:** insulin resistance, iron, diabetes, hepcidin, hepatitis c, ferritin, glucose

## Abstract

Backgrounds and aim

Besides the clinical evidence supporting insulin resistance in chronic hepatitis C (CHC) patients, the exact mechanism elucidating insulin resistance is still under discussion. The present study aimed to observe any relationship between serum hepcidin, serum iron, and insulin resistance in CHC patients.

Methodology

A total of 54 individuals were recruited in this study, assorted into group A (CHC population with diabetes) and control group B (CHC population without diabetes). Both groups were tested for serum hepcidin, iron, ferritin, and serum glycemic indices (fasting blood glucose, serum insulin, and insulin resistance). Serum parameters were compared between diabetic and non-diabetic CHC patients by using the Mann-Whitney U test. Correlation analysis was done between serum hepcidin and serum iron, serum hepcidin, and insulin resistance, and serum iron and insulin resistance by applying the Spearman correlation test.

Results

Diabetic and non-diabetic CHC populations exhibited an iron profile of chronic illness, i.e., low serum iron and hepcidin along with normal ferritin levels. Also, the diabetic and non-diabetic CHC population exhibited normal serum insulin and insulin resistance. However, the fasting serum glucose of the diabetic CHC population was higher than normal. Correlation analysis indicated a negative significant correlation (rho=-0.404, p=0.036) between serum iron and insulin resistance among the diabetic CHC population.

Conclusion

Our study could not provide any mounting evidence in favor of insulin resistance in the chronic hepatitis C population via serum iron or hepcidin. Hepatitis C virus causing diabetes mellitus may have some etiology other than iron metabolism.

## Introduction

The liver has a special role to play in iron homeostasis by influencing iron uptake, storage, transport, and mobilization from storage to maintain erythropoietic demands [1]. Quest for the regulation of iron led to the discovery of a hepatic regulatory hormone liver expressed antimicrobial peptide (LEAP-1) from plasma ultrafiltrate, which was later named hepcidin [2]. Hepcidin binds with the ferroportin (cellular iron exporter present on enterocytes, macrophages, and hepatocytes), followed by phosphorylation and clathrin-mediated internalization of hepcidin-ferroportin complexes [3]. Adequate iron stores in blood signal for increased hepatic expression of hepcidin and internalization of ferroportin, thus preventing the iron efflux from enterocytes and vice versa. However, erythropoietic and inflammatory clues can also potentially influence hepcidin production, sometimes even dominating the iron storage signals [3].

An absolute or relative deficiency of hepcidin is an etiological factor for iron overload [4]. Chronic hepatitis C virus (HCV) infection is a prevalent cause of iron overload by enhanced expression of core+1/alternate reading frame protein (ARFP) induced inhibition of hepcidin promoter and hepcidin transcription [5]. Another HCV protein, nonstructural protein 5A (NS5A) decreases the hepcidin expression by inhibiting its messenger RNA, thus increasing iron stores and its deposition in hepatocytes and macrophages [6, 7].

World health organization (WHO) has declared that hepatitis C infection is a major health issue owing to its hepatic (cirrhosis, hepatocellular carcinoma) and extrahepatic (diabetes mellitus, depression, renal disease) complications. WHO estimated the global prevalence of chronic hepatitis C (CHC) infection to be approximately 71 million in 2015. WHO also estimated that 15% of patients of CHC develop diabetes mellitus as a complication, irrespective of the degree of liver fibrosis [8].

Diabetes mellitus in CHC patients is frequently ascribed to a correlation between increased serum and hepatic iron stores and insulin resistance or direct damage to the pancreatic β cells [9]. Though many studies are supporting that iron overload is leading to insulin resistance, yet many studies have also raised the concern of raised serum ferritin levels as a marker of inflammation in CHC patients [10-12]. Though increased serum and hepatic iron stores among CHC patients are hypothetically responsible for hyperglycemia by causing insulin resistance or depletion, the actual cause of the development of diabetes mellitus in CHC patients needs to be elucidated yet [13]. 

The purpose of the present study was to fill this knowledge gap by observing the levels of serum hepcidin in CHC patients, which could give an insight into serum iron levels and the development of insulin resistance in these patients. We intended to see any correlation between serum hepcidin and iron levels with diabetes in CHC patients. Thus, the established information can trigger further experimental researches and help unleash potential therapeutic targets to reduce the disease burden in CHC patients.

## Materials and methods

This cross-sectional study was conducted at the University of Health Sciences (UHS) Lahore, Pakistan, after taking ethical approval from the Institutional Board of Advanced Studies of UHS. A total of 54 chronic hepatitis C (CHC) male patients diagnosed by polymerase chain reaction (PCR) were selected for this study by a convenience sampling technique. Selected CHC subjects were distributed in groups A and B (n=27 each), characterized by the presence and absence of diagnosed diabetes mellitus, respectively. Patients who had received blood transfusions/donations, iron supplementation, or treatment that raises blood glucose levels (corticosteroids, chemotherapy, radiotherapy, immunosuppressants, and interferons) were excluded from the study. Terminally ill CHC patients, patients with superimposed acute or chronic infections, having a family history of diabetes, and patients with diseases that could affect the interpretation of iron parameters, e.g., porphyria, thalassemia, hemochromatosis, phlebotomy, menorrhagia, anemia, occult blood loss, hematemesis, melena, hemorrhoids, and alcohol intake, etc. were also excluded. Detailed history taking and physical examination were ensured to rule out the patients that were not fitted in inclusion criteria.

Written informed consent was taken from all subjects. After 8-12 hours of overnight fast, 10 ml of venous blood was drawn to determine the iron profile (serum iron, hepcidin, and ferritin) and glucose homeostasis parameters (serum glucose and insulin). The required amount of serum as mentioned on kit details was saved in properly labeled Eppendorf Tubes®. Estimation of serum hepcidin was performed by human hepcidin (Hepc) enzyme-linked immunosorbent assay (ELISA) kit (Glory Science Co., Del Rio, USA). Serum iron was determined by a colorimetric method (Randox Laboratories Ltd., Crumlin, UK). The determination of serum ferritin was quantitatively done by using a kit manufactured by Diametra (Spello, Italy) with an automated enzyme immunoassay (EIA) analyzer, CODA®.

Serum glucose was measured by the glucose oxidase-phenol and 4 aminophenazone (GOD-PAP) method using an enzymatic colorimetric test (Human Gesellschaft for Biochemica and Diagnostica GmbH, Wiesbaden, Germany). The quantitative determination of serum insulin levels in subjects, kit (Monobind Inc., Lake Forest, USA) was used on an automated ELISA analyzer CODA®. Insulin resistance was calculated from fasting serum glucose (mmol/l) and fasting serum insulin (µ IU/ml) by homeostatic model assessment for insulin resistance (HOMA-IR) by using the following formula [14]: HOMA-IR = fasting serum glucose × (fasting serum insulin/405).

The data were analyzed using IBM SPSS version 20.0 (IBM Inc., Armonk, USA). Normal distribution of the data was checked by Shapiro-Wilk’s statistics, and if the p-value was ≤ 0.05, data was considered to be non-normally distributed. Mean ± SD is given for normally distributed quantitative variables and median with interquartile range (IQR) for non-normally distributed quantitative variables. Mann-Whitney U test was used to compare median values of non-normally distributed serum parameters among two groups. Spearman’s rho correlation (rho) was used to observe a correlation between non-normally distributed quantitative variables. A p-value of ≤ 0.05 was considered statistically significant.

## Results

The analysis of the iron profile and glucose homeostasis parameters indicated that the data is non-normally distributed. Median values of serum parameters along with interquartile ranges are given in Table 1. Median serum hepcidin and iron values are below the normal ranges among CHC patients with or without diabetes. Median blood glucose levels of CHC patients with diabetes are above the normal range. Whereas the median blood glucose levels of non-diabetic CHC patients and median serum insulin CHC patients with or without diabetes are within normal ranges (Table 1). Comparison of Iron profile and glucose homeostasis parameters by the Mann-Whitney U test indicated that the diabetic CHC population had significantly higher blood glucose levels as compared to the non-diabetic CHC population (p=0.049). Whereas iron profile, insulin, and insulin resistance were not significantly different among the two groups as assesses by the Mann-Whitney U test (Table 1).

**Table 1:** Serum levels of iron and glucose homeostasis profile among chronic hepatitis C patients, with and without diabetes CHC - chronic hepatitis C; IQR - interquartile range ^$^HOMA-IR - homeostatic model assessment of insulin resistance [15] *significant at p<0.05

Parameter	Normal range	Group A (CHC with diabetes)	Group B (CHC without diabetes)	Mann-Whitney U Test	
		Median (IQR)	Median (IQR)	U	p-value
Serum hepcidin	29 - 254 ng/ml	7.25 (6.11 - 8.37)	7.54 (6.59 - 8.65)	398.5	0.56
Serum iron	10.6 - 28.3 µmol/l	4.29 (1.79 - 7.16)	5.01 (1.79 - 9.49)	374	0.87
Serum ferritin	20 - 400 ng/ml	161 (37 - 431)	114 (60 - 201)	301	0.27
Blood glucose	70 - 100 mg/dl	110 (75 - 127)	96 (82 - 99)	251	0.049*
Serum insulin	0.7 - 9 µU/ml	5.00 (3 - 12)	7.00 (4 - 4.7)	423	0.31
HOMA-IR^$^	1.5 - 4	1.22 (0.84 - 2.85)	1.46 (0.77 - 3.25)	379	0.80

**Figure 1:**
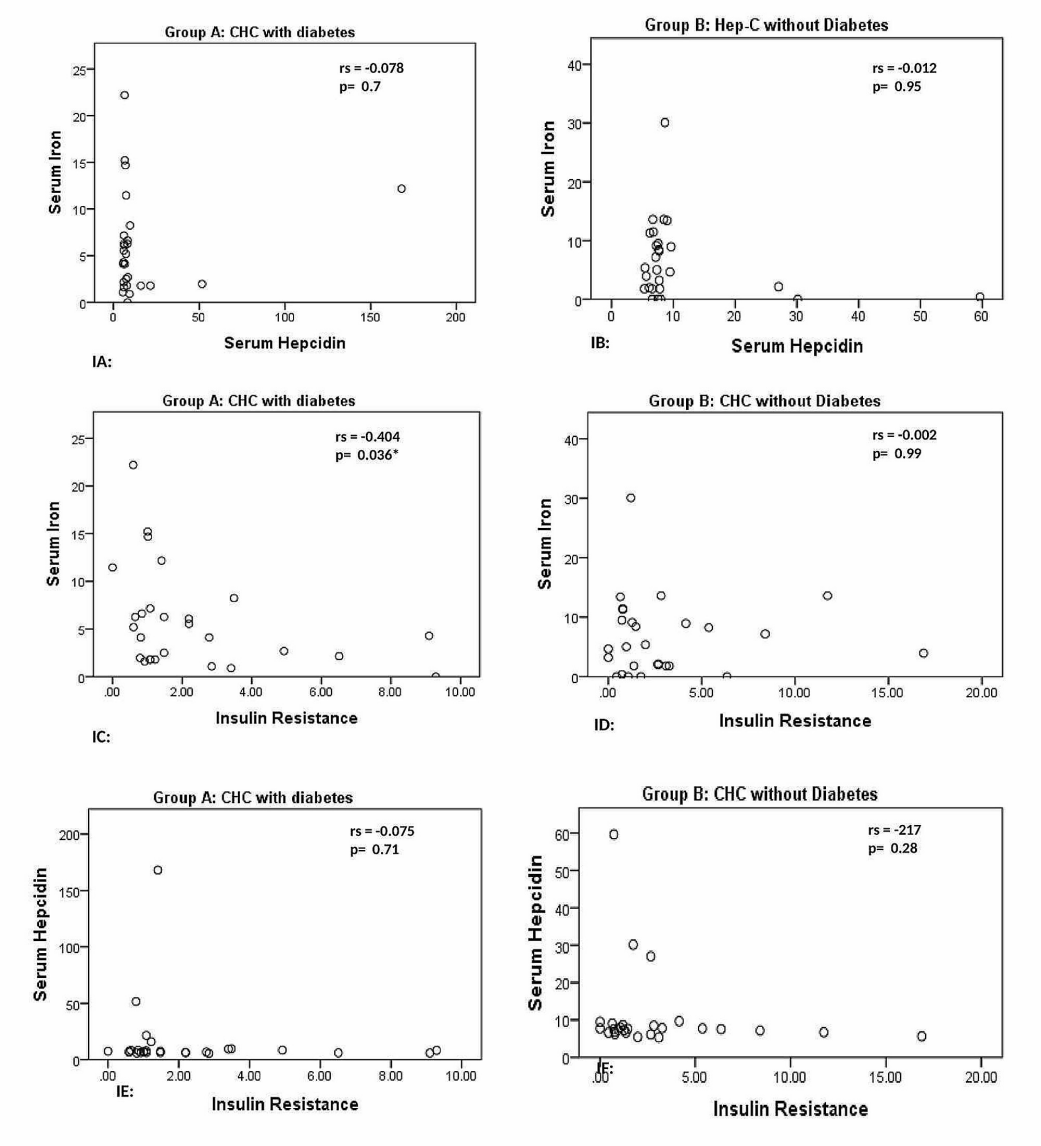
Correlations between hepcidin and iron, iron and insulin resistance, hepcidin and insulin resistance in chronic hepatitis C patients CHC - chronic hepatitis C; rs - Spearman’s rho correlation coefficient *Correlation between iron and insulin resistance among diabetic CHC patients is significant at p-value ≤ 0.05 (see graph IC)

A significant negative correlation was observed between iron and insulin resistance in CHC diabetic population (rho=-0.404, p=0.036). There was negative, non-significant correlation between hepcidin and iron (rho=-0.078, p=0.700) and hepcidin and insulin resistance (rho=-0.075, p=0.708) in CHC diabetic population (Figure 1). 

There was a negative, non-significant correlation between hepcidin and iron (rho=-0.012, p=0.953) and hepcidin and insulin resistance (rho=-0.217, p=0.276) in CHC patients without diabetes. Iron and insulin resistance (rho=-0.217, p=0.276) were also non-significantly negatively correlated in CHC patients without diabetes (Figure 1). 

## Discussion

The present study was conducted to assess the link between chronic hepatitis C (CHC) and insulin resistance via serum hepcidin and serum iron levels. For this purpose, CHC patients with and without diabetes were assorted into groups A and B, respectively. Both groups were tested for serum hepcidin, iron, ferritin levels, and serum glycemic indices. CHC patients with diabetes showed decreased serum iron and hepcidin, along with normal serum ferritin levels, which suggests iron deficiency with ongoing chronic inflammation, as earlier reported by Wians et al. In non-diabetic CHC patients, decreased serum hepcidin and iron levels were observed along with normal ferritin, again consistent with the parameters of chronic illness as supported by Wians et al. [16]. Similar results were obtained by a study conducted by Marzouk et al. on 30 CHC patients and 20 healthy volunteers as controls, showing less serum hepcidin levels along with less serum iron and raised serum ferritin levels among CHC patients [17]. Decreased levels of the iron regulatory hormone (serum hepcidin) in diabetic/non-diabetic CHC patients could be due to two reasons, i.e., decreased serum iron levels and hepatitis C virus. The Hepatitis C virus itself is notorious for decreasing serum hepcidin levels [18].

In our study, the diabetic and non-diabetic CHC population showed no insulin resistance with normal serum insulin levels. However, the fasting serum glucose levels of diabetic CHC patients were higher than the normal range. In group A diabetic CHC population, high fasting blood glucose levels with normal serum insulin levels was an important observation assuming that HCV in group A caused diabetes by some other etiological factor. Uncontrolled glucose production due to transcriptional modification of gluconeogenesis because of HCV induced endoplasmic reticulum stress could be a cause of higher than normal glucose levels in diabetic CHC patients of the current study [19]. HCV is also considered to cause diabetes by proteasome ubiquitination of insulin receptor substrates 1 and 2 (IRS1/2), by effecting insulin signaling pathways or immune-mediated stress plays [20].

To assess the relationship of hyperglycemia with CHC iron profile, correlation analysis was done between serum hepcidin and serum iron, serum iron and insulin resistance, and finally between serum hepcidin and insulin resistance in both groups. In the current study, CHC patients with diabetes exhibited a negative non-significant correlation between serum hepcidin with serum iron and serum hepcidin and insulin resistance. A positive association between the dysregulation of iron metabolism and deranged glucose parameters was reported in previous studies, which is quite different from the current study [21, 22].

In the current study, CHC diabetic group showed a negative significant correlation of serum iron with insulin resistance (IR), showing that iron can influence insulin resistance even in the absence of significant iron load, as supported by previous findings [23]. Petit et al. performed a study on 103 HCV-infected patients to access the HCV effects on hepatic iron content and IR [9]. No significant association between histological iron grading and insulin resistance was found. A negative significant correlation between serum iron and IR in group A suggests that there must be some other factor than serum iron causing insulin resistance by HCV. HOMA-IR showed a significant positive correlation with hepatic iron levels in a study conducted by Sumida et al. [24]. The results of that study were significantly different from the current study; the reason could be that in the present study, liver iron grading was not performed.

HCV is a culprit to lead to diabetes, or the diabetic population has more incidences to get hepatitis C infection is still a mystery to be solved, though it is still said without any ambiguity that HCV has some direct and indirect roles in diabetes. The small sample size was a big limitation of our study as certain parameters showed a negative, non-significant correlation, e.g., between serum hepcidin and serum iron parameters in CHC diabetics and CHC patients without diabetes. If the sample size would be bigger, there could be more chances of turning non-significant into significant correlations. Another big limitation was the lack of hepatic iron grading, as hepatic iron grading could be a revealing factor to access iron loads and potential indirect testing parameters of insulin resistance. Future studies should be conducted with a larger sample size to establish significant correlations between parameters, if any. Studies should also be conducted to access hepatic iron grading and messenger ribonucleic acid (mRNA) hepcidin levels in relation to diabetes mellitus among CHC patients.

## Conclusions

The present study was designed to assess the relationship between serum iron and insulin resistance, linking serum hepcidin levels in the chronic hepatitis C population. This study could not provide any mounting evidence in favor of insulin resistance in the CHC population via serum iron and hepcidin levels. Higher than normal fasting blood glucose with normal serum insulin levels among the diabetic CHC population of this study favors pathogenesis of HCV induced diabetes mellitus by some other etiological factor. The current study results imply that HCV induced diabetes might have some cause other than serum iron metabolism (as proposed by previous literature such as reactive oxygen species induced by hepatitis C, insulin signaling defects, or steatosis).

## References

[1] Daher R, Manceau H, Karim Z (2017). Iron metabolism and the role of the iron-regulating hormone hepcidin in health and disease. Presse Méd.

[2] Krause A, Neitz S, Magert HJ, Schulz A, Forssmann WG, Schulz-Knappe P, Adermann K (2000). LEAP-1, a novel highly disulfide bonded human peptide, exhibits antimicrobial activity. FEBS Lett.

[3] Maliken BD, Nelson JE, Kowdley KV (2011). The hepcidin circuits act: balancing iron and inflammation. Hepatology.

[4] Casu C, Nemeth E, Rivella S (2018). Hepcidin agonists as therapeutic tools. Blood.

[5] Kotta-Loizou I, Vassilaki N, Pissas G (2013). Hepatitis C virus core+ 1/ARF protein decreases hepcidin transcription through an AP1 binding site. J Gen Virol.

[6] Liu Y, Xiao X, Cheng D, Jiang Y, Gong G (2011). HCV NS5A protein down-regulates hepcidin gene expression and increases hepatic intracellular iron storage (in Chinese). Zhonghua gan zang bing za zhi.

[7] Price L, Kowdley KV (2009). The role of iron in the pathophysiology and treatment of chronic hepatitis C. Can J Gastroenterol.

[8] (2018). Guidelines for the care and treatment of persons diagnosed with chronic hepatitis C virus infection. http://www.who.int/hepatitis/publications/hepatitis-c-guidelines-2018/en/.

[9] Petit J-M, Bour J-B, Galland-Jos C (2001). Risk factors for diabetes mellitus and early insulin resistance in chronic hepatitis C. J Hepatol.

[10] Sachinidis A, Doumas M, Imprialos K, Stavropoulos K, Katsimardou A, Athyros VG (2020). Dysmetabolic iron overload in metabolic syndrome. Curr Pharm Des.

[11] Britton L, Bridle K, Reiling J (2018). Hepatic iron concentration correlates with insulin sensitivity in nonalcoholic fatty liver disease. Hepatol Commun.

[12] Lecube A, Hernandez C, Simo R (2009). Glucose abnormalities in non‐alcoholic fatty liver disease and chronic hepatitis C virus infection: the role of iron overload. Diabetes Metab Res Rev.

[13] Kralj D, Jukić LV, Stojsavljević S, Duvnjak M, Smolić M, Čurčić IB (2016). Hepatitis C virus, insulin resistance, and steatosis. J Clin Transl Hepatol.

[14] Marder W, Khalatbari S, Myles JD (2013). The peroxisome proliferator activated receptor-c pioglitazone improves vascular function and decreases disease activity in patients with rheumatoid arthritis. J Am Heart Assoc.

[15] Eslam M, Kawaguchi T, Del Campo J, Sata M, Abo‐Elneen Khattab M, Romero‐Gomez M (2011). Use of HOMA‐IR in hepatitis C. J Viral Hepat.

[16] Wians Jr FH, Urban JE, Keffer JH, Kroft SH (2001). Discriminating between iron deficiency anemia and anemia of chronic disease using traditional indices of iron status vs transferrin receptor concentration. Am J Clin Pathol.

[17] Marzouk HA, Zayed NA, Al-Ansary M (2013). Hepcidin levels in Egyptian patients with chronic hepatitis C and the effect of anti-viral therapy. World Appl Sci J.

[18] El Lehleh AM, El Shazly RA, Hamza RR (2017). Study of serum hepcidin in patients with chronic hepatitis C. Menoufia Med J.

[19] Hatting M, Tavares CD, Sharabi K, Rines AK, Puigserver P (2018). Insulin regulation of gluconeogenesis. Ann N Y Acad Sci.

[20] Naing C, Mak JW, Ahmed SI, Maung M (2012). Relationship between hepatitis C virus infection and type 2 diabetes mellitus: meta-analysis. World J Gastroenterol.

[21] Ali ST, Mohamed NA (2019). Inadequate serum hepcidin levels in chronic hepatitis C infection-induced type 2 diabetes mellitus. Sci J Al-Azhar Med Fac Girls.

[22] Varghese J, James JV, Anand R (2020). Development of insulin resistance preceded major changes in iron homeostasis in mice fed a high-fat diet. J Nutr Biochem.

[23] Zafar U, Qureshi HJ, Karim A (2011). Insulin resistance and serum parameters of iron status in type 2 diabetics. PJP.

[24] Sumida Y, Kanemasa K, Fukumoto K, Yoshida N, Sakai K (2007). Hepatic iron accumulation may be associated with insulin resistance in patients with chronic hepatitis C. Hepatol Res.

